# Association of shared decision-making with type of breast cancer surgery: a cross-sectional study

**DOI:** 10.1186/1472-6963-10-48

**Published:** 2010-02-23

**Authors:** Myung Kyung Lee, Dong Young Noh, Seok Jin Nam, Se Hyun Ahn, Byeong Woo Park, Eun Sook Lee, Young Ho Yun

**Affiliations:** 1 Division of Cancer Control, Research Institute for National Cancer Control and Evaluation, National Cancer Center, Goyang, Gyeonggi, Korea; 2 Department of Surgery, Seoul National University College of Medicine, Seoul, Korea; 3 Department of Surgery, Samsung Medical Center, Sungkyunkwan University School of Medicine, Seoul, Korea; 4 Department of Surgery, Asan Medical Center, University of Ulsan College of Medicine, Seoul, Korea; 5 Department of Surgery, Yonsei University Medical Center, Seoul, Korea; 6 Branch of Breast Cancer, Research Institute, National Cancer Center, Goyang, Gyeonggi, Korea

## Abstract

**Background:**

Although some studies examined the association between shared decision-making (SDM) and type of breast cancer surgery received, it is little known how treatment decisions might be shaped by the information provided by physicians. The purpose of this study was to identify the associations between shared decision making (SDM) and surgical treatment received.

**Methods:**

Questionnaires on SDM were administered to 1,893 women undergoing primary curative surgery for newly diagnosed stage 0-II localized breast cancer at five hospitals in Korea. Questions included being informed on treatment options and the patient's own opinion in decision-making.

**Results:**

Patients more likely to undergo mastectomy were those whose opinions were respected in treatment decisions (adjusted odds ratio, aOR), 1.40; 95% confidence interval (CI), 1.14-1.72) and who were informed on chemotherapy (aOR, 2.57; CI, 2.20-3.01) or hormone therapy (aOR, 2.03; CI, 1.77-2.32). In contrast, patients less likely to undergo mastectomy were those who were more informed on breast surgery options (aOR, 0.34; CI, 0.27-0.42). In patients diagnosed with stage 0-IIa cancer, clinical factors and the provision of information on treatment by the doctor were associated with treatment decisions. In patients diagnosed with stage IIb cancer, the patient's opinion was more respected in treatment decisions.

**Conclusion:**

Our population-based study suggested that women's treatment decisions might be shaped by the information provided by physicians, and that women might request different information from their physicians based on their preferred treatment options. These results might need to be confirmed in other studies of treatment decisions.

## Background

Patient-physician communication regarding treatment decisions is a poorly understood area of cancer care. In recent years, patients have wanted more information about their diseases and greater involvement in treatment decisions [1,2]. Since shared decision making (SDM) may result in increased compliance and better health outcomes [3], it is strongly advocated [4].

Previous studies have explored many aspects of patient-physician communications, including how they relate to actual surgical treatment [5-8], the nature of patient decision-making preferences [9] and what influences these preferences [10], the fulfillment of patients' preferred decisional roles [9-11] and their outcomes (such as satisfaction or regret) [11,12], and the use of decision aids[13]. In these situations, however, it is little known whether the physician or patient makes the primary treatment decision, whether these decisions depend on disease stage, and whether the provision of information by the physician on topics such as treatment options and their side effects, associated course of recovery, and the likelihood of recurrence, affects treatment decisions. Understanding the effects of SDM could lead to better care of cancer patients [14-16].

We conducted a large population-based survey of women with breast cancer to test our hypothesis that patient surgical preferences are influenced by the type of information provided by their physicians. The study aimed to determine (1) the prevalence of various components of shared decision making, (2) the association of SDM with surgical treatment, and (3) whether personal opinion or information provided by the physician played a bigger role in the patient's surgical decision when patient-physician communication was stratified by disease stage.

## Methods

### Study Population

We used 5 hospital-based breast cancer registries in Korea to identify women who had undergone primary curative surgery for breast cancer between 1993 and 2002. The registries contained information about tumor stage, type of surgery, history of cancer therapy, and other clinical characteristics, such as estrogen and progesterone receptor status, menopausal status at diagnosis, recurrence, and date since surgery. Patients were considered potentially eligible if they 1) had a diagnosis of stage 0 to II breast cancer and tumor size ≤ 5 cm, 2) had no other history of cancer, 3) both breast-conserving surgery and mastectomy were treatment options and 4) were alive at the time of the survey. From a total of 10,796 registered women, we identified 8,370 who were potentially eligible. Of those, we were able to contact 4,126. The most frequent reason for contact failure was a change of address or telephone number. Of the women contacted, 30.0% refused to participate. The most frequent reasons for refusal were that the survey was inconvenient and that it took too long to complete. Of the 2,888 women who agreed to participate, most (80.3%) filled out and returned the questionnaire. After reviewing the questionnaires for completeness, we were left with a total study population of 1,893 subjects. The median follow-up time after surgery was 49 months (range, 24-133 months).

### Data Collection

From May to September 2004, potentially eligible patients were contacted by telephone, and those who agreed to participate were sent a questionnaire with consent forms and a postage-paid return envelope. Subjects who did not return the questionnaire within 1 month received a reminder card and a telephone call. Multiple attempts were made to contact the remaining potential subjects by postcard or telephone. The institutional review board of the National Cancer Center reviewed and approved the protocol.

### Measures

The main dependent variable was the type of surgical treatment, either mastectomy or BCS, obtained from the clinical database. The principal independent variables were various patient-reported SDM experiences; patient sociodemographic variables, including age, education level, marital status, religion, household income, residential area (metropolitan vs. rural), size of household, and employment status; and clinical factors, including comorbidities, menopausal status, estrogen and progesterone receptor status, and tumor stage. We measured the SDM as two dimensions: being informed of alternative treatments and being patient's own opinion respected. The following questions, based on earlier studies, were used to evaluate SDM [5-10,17]. (1) Was your opinion respected before your surgical treatment decision? This item was rated on a 5-point Likert scale ranging from "strongly agree" (1) to "strongly disagree" (5). "Strongly agree" and "agree" were coded as "yes", and "neither agree nor disagree", "disagree", and "strongly disagree" were coded as "no". (2) Did your doctors provide information on breast surgery options (e.g., the techniques, consequences, benefits and drawbacks of each option) before your surgical treatment decision? (3) Did your doctors provide information on radiotherapy (e.g., whether it was mandatory, as well as its consequences, benefits and drawbacks) before your surgical treatment decision? (4) Did your doctors provide information on chemotherapy (e.g., whether it was mandatory, as well as its consequences, benefits and drawbacks) before your surgical treatment decision? (5) Did your doctors provide information on hormone therapy (e.g., whether it was mandatory, as well as its consequences, benefits and drawbacks) before your surgical treatment decision? These questions were posed to all women. The response category was "yes" or "no". Feasibility and comprehensibility of the full survey instrument--including SDM questions, and socio-demographic and clinical characteristics--were pre-tested on 15 breast cancer survivors in an outpatient clinic of the Korean National Cancer Center. Pretesting did not change the survey instrument, but an independent validation study was not performed.

### Data Analysis

All analyses were performed by weighting the data with the number of total eligible breast cancer survivors to ensure that our estimates were representative. To adjust for the differences observed between respondents and non-respondents, we used the inverse probability of response weighting approach described by Robins and colleagues [18,19]. In this approach, data for each survivor are further weighted according to the reciprocal of the conditional probability of being a respondent given all clinical variables (i.e., age and tumor stage at diagnosis, tumor size, time since surgery, type of surgery, type of adjuvant therapy, and hormone receptor status of tumor). These "adjustment" weights ensure that the effect of each response is the same in the adjusted population as in the original eligible population. The range of weights was 2.07-7.64. We evaluated the balance on covariates within propensity score quintiles in the adjusted population to confirm the accuracy of the model [20]. We also trimmed the adjustment weights to reduce the effects of influential observations on the overall results by minimizing the mean squared error [21]. These methods control only for observed characteristics; after adjustment, respondents may differ from non-respondents in unobserved characteristics. Nevertheless, these methods allow for better control of potential biases between respondents and non-respondents than methods that include only respondent data.

We computed univariate regressions of the effects of each of the dichotomously categorized SDM variables and other potential indicators upon receiving a mastectomy. We used multiple logistic regression analysis with stepwise selection to identify the best subset of independent variables for predicting surgery type. Furthermore, we stratified patients by disease stage (i.e., ranging from stages 0-IIa to IIb) and performed separate multiple logistic regression analyses to identify the subset of patients who would best illustrate the variables influencing surgical decisions according to disease stage. The significance level for entering an effect into the multiple logistic regression models by the stepwise method was .05. Results of multivariate logistic regression models were expressed as odds ratios (ORs) relative to subjects' undergoing a mastectomy. We considered *P *< .05 as statistically significant, and all statistical tests were two-sided and performed using SAS Version 9.2 (SAS Institute, Inc., Cary, NC).

## Results

Compared with patients responding to the questionnaire, a larger proportion of non-respondents were ≥ 65 yr old (62.5% *v *57.8%, *P *< .0001), had advanced (≥ stage IIb) disease (28.4% *v *25.7%, *P *< .03), underwent surgery ≥ 5 yr ago (54.1% *v *36.9%, *P *< .0001), had received mastectomies (71.2% *v *64.4%, *P *< .0001) and hormonal therapy (57.2% *v *51.5%, *P *< .0001), had not received chemotherapy (59.6% *v *64.8%, *P *< .0001), and had estrogen- (61.0% *v *53.5%, *P *< .0001) or progesterone-sensitive tumors (59.6% *v *49.2%, *P *< .0001). After further weighting according to inverse propensity scores for being a respondent, the respondent population was made to have distribution on these variables similar to the distributions in the full population.

### Patient Characteristics (Table 1)

Mean patient age at diagnosis was 48.6 years. Table 1 lists their socio-demographic and clinical characteristics.

**Table 1:** Demographic and clinical characteristics of patients who responded to our questionnaire

	**No**.	% of patients (n = 1,893)
**Variables at the time of initial decision making**		
Age at diagnosis, yr		
<65	1780	94.0
≥65	113	6.0
Mean (SD)		48.6 (9.4)
Educational attainment		
≤high school	1329	70.6
college or higher	554	29.4
Marital status		
no spouse	286	15.2
with spouse	1599	84.8
Having a religion		
no	282	15.0
yes	1600	85.0
Monthly household income		
<US$3000	903	55.5
≥US$3000	723	44.5
Residential area		
rural	188	10.0
metropolitan	1696	90.0
No. of family members		
<3	1345	71.0
≥3	548	29.0
Menopausal status at diagnosis		
pre	1156	61.1
post	737	38.9
Employment status at diagnosis		
no	815	44.9
yes	1000	55.1
Stage		
0-IIa	1518	80.2
IIb	375	19.8
Estrogen receptor		
negative	805	42.5
positive	1088	57.5
Progesterone receptor		
negative	844	44.6
positive	1049	55.4
Comorbidity		
0	963	50.9
≥1	930	49.1
Type of surgery		
mastectomy	1274	67.3
BCS	619	32.7
Received radiotherapy	721	38.1
Received chemotherapy	1190	62.9
Received hormone therapy	1070	56.5

Abbreviations: SD, standard deviation; BCS, breast-conserving surgery

### Patients' self-reported SDM variables

Overall, most women reported that that their opinions were respected in surgical treatment decisions (88.5%), that their doctor had informed them of surgical treatment options (89.1%), and on radiotherapy (84.2%), chemotherapy (78.5%), and hormonal therapy (66.2%).

### Factors associated with type of breast cancer surgery--univariate analyses (Table 2)

In univariate analyses, age, educational level, monthly household income, residential area, comorbidity, menopausal status, estrogen receptor status, and disease stage at the time of initial decision making were associated with type of surgery received (mastectomy versus BCS). Provision of information on surgery options by the doctors was associated with receiving BCS, whereas being patient's opinion respected and doctors' providing information on chemotherapy or hormone therapy were associated with undergoing a mastectomy.

**Table 2:** Univariate analyses* of factors associated with type of surgery

				Mastectomy (vs. BCS) N = 1,893	
				%	OR (95% CI)
**Predisposing factors at the time of initial decision making**					
Age at diagnosis,					
<65				64.9	1(referent)
≥65				71.0	1.70(1.32-2.19)
Educational level					
high school or less				67.2	1(referent)
college or higher				61.6	0.80(0.72-0.90)
Marital status					
no spouse				66.2	1(referent)
married				65.2	0.93(0.81-1.08)
Having a religion					
no				65.8	1(referent)
yes				65.2	0.99(0.86-1.14)
Monthly income					
<US$3000				68.6	1(referent)
≥US$3000				61.8	0.69(0.63-0.77)
Residential area					
rural				70.2	1(referent)
metropolitan area				64.7	0.78(0.65-0.94)
Number of adults living together					
<3				65.6	1(referent)
≥3				64.5	0.96(0.85-1.07)
Employment status					
unemployed				64.6	1(referent)
employed				65.2	1.07(0.96-1.19)
Comorbidities					
none				63.6	1(referent)
≥1				66.8	1.17(1.05-1.29)
Menopausal status					
premenopausal				63.5	1(referent)
postmenopausal				68.0	1.17(1.04-1.30)
Estrogen receptor					
negative				65.8	1(referent)
positive				62.9	0.88(0.80-0.98)
Progesterone receptor					
negative				65.1	1(referent)
positive				65.5	1.00(0.90-1.11)
Stage					
0-IIa				61.5	1(referent)
IIb				84.3	3.68(3.11-4.35)
**Shared decision making factors**					
Being patient's opinion respected in surgical treatment decision					
no				62.7	1(referent)
yes				73.3	1.48(1.24-1.76)
**Physician's giving information**					
Breast surgery option					
no				74.5	1(referent)
yes				63.7	0.59(0.49-0.71)
Using radiotherapy (ref. no)					
no				67.1	1(referent)
yes				65.9	0.95(0.82-1.09)
Using chemotherapy (ref. no)					
no				44.0	1(referent)
yes				72.0	3.17(2.79-3.59)
Using hormone therapy (ref. no)					
no				49.1	1(referent)
yes				73.4	2.41(2.15-2.69)

Abbreviation: BCS, breast-conserving surgery; CI, confidence interval

* All analyses weighted to account for differential selection by clinical factors of non-response.

### Factors associated with type of breast cancer surgery--multivariate logistic regression analysis (Table 3)

Multivariate regression analysis indicated that women were more likely to undergo mastectomies than BCS if they were premenopausal, diagnosed with stage IIb breast cancer, had comorbidities, and were informed on the use of chemotherapy or hormone therapy by their doctors. Conversely, women were less likely to undergo mastectomies if they had estrogen-sensitive tumors, or if they were informed on breast surgery options by their doctors.

**Table 3:** Multivariate analysis* of factors associated with surgical treatment

	Mastectomy (vs. BCS) N = 1,893 OR* (95% CI)
**Predisposing factors**	
Comorbidities (ref. none)	1.19(1.05-1.34)
Premenopausal (ref. postmenopausal)	1.26(1.11-1.43)
Estrogen receptor (ref: negative)	0.81(0.72-0.91)
Stage, IIb (ref: 0-IIa)	2.55(2.15-3.02)
**Shared decision making**	
Being patient's opinion respected in treatment decision (ref. no)	1.40(1.14-1.72)
**Physician's giving information**	
Breast surgery option (ref. no)	0.34(0.27-0.42)
Using chemotherapy (ref. no)	2.57(2.20-3.01)
Using hormone therapy (ref. no)	2.03(1.77-2.32)

Abbreviation: BCS, breast-conserving surgery; CI, confidence interval

* All analyses weighted to account for differential selection by clinical factors of non-response.

** Results of logistic regression with stepwise selection methods, whose covariates were age at diagnosis, educational level, monthly income, residential area, menopausal status at diagnosis, comorbidity, estrogen receptor, disease stage, patient's participation in treatment decision, physician's giving information on surgery, physician's giving information on chemotherapy, and physician's giving information on hormone therapy, all of which were significant in univariate analyses.

### Factors associated with type of breast cancer surgery according to disease stage--multivariate logistic regression analyses (Table 4)

In multivariate logistic regression analysis, factors associated with type of treatment varied with tumor stage. Women with early stage (0-IIa) disease were more likely to undergo mastectomies than BCS if they had higher stage disease, or had received information on the use of chemotherapy or hormone therapy. They were less likely to undergo mastectomies if they had estrogen-sensitive tumors or had been informed on breast surgery options. Women diagnosed with more advanced disease (IIb) were less likely to undergo mastectomies if they were more highly educated and were more likely to undergo mastectomies if they were older, premenopausal, had more comorbidities, had relied more on their own opinions in making treatment decisions, or had been informed on the use of hormone therapy by their doctors.

**Table 4:** Multivariate analyses* of factors associated with surgical treatment according to disease stage

		Mastectomy (ref. BCS) OR (95% CI)	
		Stage 0-IIa** n = 1,518	Stage IIb*** n = 375

**Patients' predisposing factors**			
Age at diagnosis, ≥65 (ref:< 65)		NA	5.63(1.80-7.60)
Comorbidities (ref. none)		NA	2.07(1.39-3.09)
College or higher (ref. high school or less)		NA	0.60(0.43-0.85)
Premenopausal (ref. Postmenopausal)		NA	1.43(1.25-1.63)
Estrogen receptor, positive (ref: negative)		0.79(0.69-0.89)	NA
Higher stage****		1.25(1.13-1.38)	NA
**Shared decision making**			
Being patient's opinion respected in treatment decision (ref. no)		NA	5.43(3.48-8.46)
**Physician's giving information**			
Breast surgery option (ref. no)		0.36(0.28-0.45)	NA
Using chemotherapy (ref. no)		2.79(2.37-3.29)	NA
Using hormone therapy (ref. no)		1.94(1.67-2.24)	2.69(1.92-3.75)

Abbreviation: BCS, breast-conserving surgery; NA, Not available; CI, confidence interval

*All analyses weighted to account for differential selection by clinical factors of non-response.

** Results of logistic regression with stepwise selection methods whose covariates were age at diagnosis, monthly income, residential area, comorbidity, estrogen receptor status, disease stage (continuous type), patient's participation in treatment decision, physician's giving information on surgery, physician's giving information on chemotherapy, and physician's giving information on hormone therapy, which were significant in univariate analyses.

**The results of logistic regression with stepwise selection methods, whose covariates were age at diagnosis, educational level, marital status, comorbidity, menopausal status at diagnosis, progesterone receptor status, patient's participation in treatment decision, physician's giving information on chemotherapy, and physician's giving information on hormone therapy, all of which were significant in univariate analyses.

**** Stage 0, I, and IIa entered into model as continuous type such as 0, 1, and 2 in early stage disease.

## Discussion

Our study focused on a relatively young and predominantly pre-menopausal group of women with early-stage breast cancer who had a relatively high overall rate of mastectomy. Few studies have explored breast surgery in this type of population, and there are wide variations in mastectomy rates both within and between different countries.

Our results suggest that patients might prefer mastectomy whereas surgeons might prefer breast conservation. Recent reports have indicated that when fully informed about the risks and benefits of various surgery options, a significant proportion of patients will actively choose mastectomy [22]. We studied women with stage 0-II breast cancer to examine the associations between SDM and treatment decisions according to disease stage and to determine whether the patient or physician plays the primary role in determining surgical options. Treatment decisions made by women with early-stage breast cancer may be more affected by the surgeon's providing of information, whereas decisions made by women with advanced stage breast cancer may be more dependent on their own opinions. Although most patients want to share in the decision making process [1,2], our findings suggest that treatment decisions are dependent on disease stage. For example, patients with stage IIb breast cancer may play a more collaborative role in treatment decisions than those with early-stage breast cancer, and a collaborative role may be more associated with mastectomy than BCS. Previous studies [11,23] have reported that women who reported being most active in decision making were less likely than more passive women to undergo BCS, suggesting that women with more autonomy are more likely to choose a procedure perceived as more definite [11,23]. This autonomy may be associated with maximizing patient outcomes [11]. Because patients believed that mastectomy was clinically superior, those who played a greater role in decision making were more likely to undergo mastectomy [24]. Patients may prefer mastectomy over BCS because of concerns about recurrence of disease, recovery from surgery, and side effects of radiation treatment [25]. Women with stage IIb breast cancer may consider their doctors' opinions as important factors in decision making. However, women uncertain about treatment decisions can ask questions regarding the consequences of surgery and the possibility of recurrence. In addition, some surgeons may continue to believe that mastectomy is clinically superior to BCS, particularly because of the lower risk of local disease recurrence [25]. The underlying thoughts of both the doctor and the more autonomous patient during the decision-making process may lead to consensus on the appropriate surgical treatment, and this collaboration may lead to the decision for mastectomy.

In contrast, better informed early-stage breast cancer patients tend to choose BCS over mastectomy [26]. Knowledge about the benefits of mastectomy vs. BCS was the strongest predictor of BCS [27]. Moreover, we found that clinical factors may be important in treatment decisions made by women with early-stage breast cancer. Objective factors, such as tumor stage and estrogen receptor status, together with adequate information on breast surgery options, chemotherapy, and hormone therapy may be associated with treatment decisions. Due to the workload of medical teams and the lack of time, it may be difficult for physicians to spend time with breast cancer patients who have a good prognosis, in order to reach a decision on treatment. In addition, being given a choice of treatments may be associated with increased emotional distress [28]. Because patients put value on the information regardless of their preferred decision-making style [1,2], providing sufficient information may be important to early-stage breast cancer patients. We found that collaboration in decision making may be more feasible among stage IIb than among early-stage breast cancer patients.

Our finding, that premenopausal women with advanced disease were more likely to receive mastectomy and women with hormonally sensitive early stage disease were more likely to receive BCS, is consistent with other studies [29,30] and treatment guidelines [31,32].

We found that among women with stage IIb tumors, those who were older or less educated were more likely to undergo mastectomy than BCS, in agreement with previous results [33-35]. One previous study suggested that better educated and better informed older women were more likely to undergo BCS [36]. Although undergoing a mastectomy correlates with a patient's involvement in decision making, mastectomy had been found to depend on physician recommendations [35]. Physicians may take a greater initiative in the decision making process for older patients and those who have less knowledge about breast cancer [35,37]. Indeed, we found that breast cancer knowledge was the strongest predictor of BCS [27]. These observations are consistent with other reports regarding the influence of age and education on the decision making process in breast cancer patients [38,39].

When making a decision for patients with stage IIb breast cancer, a surgeon's sensitivity to patient personal concerns and anxiety about poor prognosis caused by comorbidity would likely make patients more willing to follow the surgeon's recommendation or to choose mastectomy over BCS.

The association of provision of information regarding chemotherapy and hormone therapy with a greater likelihood of undergoing mastectomy may be due to a desire for a perceived long-term effect. For patients with hormone-sensitive tumors, mastectomy followed by tamoxifen or aromatase inhibitor for postmenopausal patients may be the safest option [40,41]. Although the interpretation of information within patients' social contexts may lead to different surgical choices [42] and the decision-making process gets more complex, the decision-making pattern may be consistent when physicians provide their patients with a summary of available treatment options, together with information about the associated risks and benefits.

Our study had several limitations. Due to the retrospective nature of this study, a considerable amount of time had elapsed between the treatment and the beginning of the study; therefore, our results may have been influenced by recall bias. Second, our sample had a low response rate, with respondents differing from non-respondents. After weighting the inverse probability of response, however, the two groups were similar. Although we made adjustments only for observed characteristics, the method controls for any potential bias between respondents and non-respondents. Third, we did not use standard tools to assess information needs and decision-making strategies. Instead, we designed our own tool to assess the degree of shared decision making. The potential flaws in the questionnaire are respected in some of the observed outcomes. Thus, a validated tool must be used to assess the topic of this study. Furthermore, we did not include separate SDM questions to determine if patients were told that the treatment was mandatory, and whether the patients were informed of the consequences, benefits and drawbacks of each option. We presume that patients responded "yes" if they considered one of or more the sub-portions to be correct, whereas patients responded "no" if they considered none of the sub-portions to be correct. It may have been helpful to determine the physicians' responses, as recalled by the patient or the physician. Also the type of information provided by the physicians would differ for patients who received BCS versus those who received mastectomy. Fourth, we assessed SDM by patients' self-report and lack of information about physician opinion or perception of the encounter. However, we measured an essential part of the SDM process--being informed on treatment options and accommodation of patients' values--but measuring multifactorial decision-making process is difficult [43]. We used self reports because patient-reported outcome is important and patients may view SDM differently from health professionals [44]. We showed that women who report having been respected in the decision-making process more frequently received mastectomies than BCS, and women who report having been very informed about their options for surgery more frequently received BCS than mastectomies. This study was a cross-sectional survey and also we did not obtain any data about the kind of information women request from their doctors or their preferences prior to making a decision. We should interpret the set of findings with care, however, as it is possible that physicians provide more information about surgery to women who eventually get BCS, and that this might influence patient's decision toward BCS, and another finding could be that women in this population tend to prefer mastectomy and thus report having their options respected when get mastectomy.

## Conclusions

Our population-based study suggested that women's treatment decisions might be shaped by the information provided by physicians, and that women might request different information from their physicians based on their preferred treatment options. These results should be confirmed in other studies of treatment decisions.

## Competing interests

The authors declare that they have no competing interests.

## Authors' contributions

Myung Kyung Lee and Young Ho Yun conceived of the study, participated in the design of the study, data collection, performed the statistical analysis, and drafted the manuscript. Dong Young Noh, Seok Jin Nam, Se Hyun Ahn, Byeong Woo Park, and Eun Sook Lee participated in its design and coordination and helped to draft the manuscript. All authors read and approved the final manuscript.

## Pre-publication history

The pre-publication history for this paper can be accessed here:

http://www.biomedcentral.com/1472-6963/10/48/prepub
